# Autotaxin Inhibition with IOA-289 Decreases Breast Tumor Growth in Mice Whereas Knockout of Autotaxin in Adipocytes Does Not

**DOI:** 10.3390/cancers15112937

**Published:** 2023-05-26

**Authors:** Xiaoyun Tang, Andrew J. Morris, Marcel A. Deken, David N. Brindley

**Affiliations:** 1 Cancer Research Institute of Northern Alberta, Department of Biochemistry, University of Alberta, Edmonton, AB T6G 2S2, Canada; xtang2@ualberta.ca; 2 Central Arkansas Veterans Affairs Healthcare System and University of Arkansas for Medical Sciences, 4301 W. Markham St., Little Rock, AR 72205, USA; ajmorris@uams.edu; 3 iOnctura BV, Gustav Mahlerplein 102, 1082 MA Amsterdam, The Netherlands; m.deken@ionctura.com

**Keywords:** autotaxin (ATX), breast tumors, cytokines, CD8α^+^-T cells, inflammation, lysophosphatidic acid (LPA), tumor microenvironment

## Abstract

**Simple Summary:**

Breast cancer cells produce negligible quantities of autotaxin. We hypothesized that the autotaxin produced by inflamed breast adipocytes adjacent to breast tumors provides a major source of autotaxin secretion that drives breast tumor progression and the loss of treatment efficacy. We tested this hypothesis by using mice with an adipocyte-specific knock out of autotaxin. This knockout did not decrease the growth of E0771 tumors in syngeneic C57BL/6 mice or the growth and lung metastasis of spontaneous breast tumors in MMTV-PyMT mice. Despite this, the inhibition of autotaxin with IOA-289 decreased the growth of E0771 tumors in the mice. This demonstrates that cells other than adipocytes are responsible for promoting breast tumor growth.

**Abstract:**

Breast cancer cells produce negligible quantities of autotaxin. Instead, previous work indicated that adipocytes in the inflamed adipose tissue adjacent to breast tumors are a major source of autotaxin secretion that drives breast tumor growth, metastasis, and the loss of efficacy for chemotherapy and radiotherapy. To test this hypothesis, we used mice with an adipocyte-specific knock out of autotaxin. The lack of autotaxin secretion from adipocytes failed to decrease the growth of orthotopic E0771 breast tumors in syngeneic C57BL/6 mice and the growth and lung metastasis of spontaneous breast tumors in MMTV-PyMT mice. However, the inhibition of autotaxin with IOA-289 decreased the growth of E0771 tumors, indicating that another source of autotaxin is responsible for tumor growth. Tumor-associated fibroblasts and leukocytes produce the majority of autotoxin transcripts in the E0771 breast tumors, and we hypothesize that they are the main sources of ATX that drive breast tumor growth. Autotaxin inhibition with IOA-289 increased the numbers of CD8α^+^-T-cells in the tumors. This was accompanied by decreases in the concentrations of CXCL10, CCL2, and CXCL9 in the plasma and LIF, TGFβ1, TGFβ2, and prolactin in the tumors. Bioinformatics analysis of human breast tumor databases showed that autotaxin *(ENPP2)* is expressed mainly in endothelial cells and fibroblasts. Autotaxin expression correlated significantly with increases in IL-6 cytokine receptor ligand interactions, signaling by LIF, TGFβ, and prolactin. This confirms the relevance of results from autotaxin inhibition in the mouse model. We propose that inhibiting autotaxin activity that is derived from cells presenting breast tumors such as fibroblasts, leukocytes, or endothelial cells changes the tumor micro-environment in such a way as to inhibit tumor growth.

## 1. Introduction

The secreted enzyme, autotaxin (ATX), produces lysophosphatate (lysophosphatidic acid, LPA), which signals through six G protein-coupled receptors. LPA signaling is terminated by lipid phosphate phosphatases, which degrade LPA. Signaling by LPA facilitates wound healing by increasing the migration and division of cells needed for tissue repair and angiogenesis [1,2]. After the injury, ATX transcription and secretion are stimulated following the release of inflammatory cytokines [3]. LPA then increases the synthesis of COX-2 and other inflammatory mediators, which causes additional ATX secretion in a feed forward cycle [1]. LPA increases innate immune responses and promotes lymphocyte extravasation and conversion of monocytes to macrophages, which maintains immune homeostasis. Inflammation resolves when the tissue is repaired and ATX secretion decreases. If inflammation is not resolved, chronic activation of ATX-LPA-inflammatory signaling becomes maladaptive in pulmonary fibrosis, cirrhosis, rheumatoid arthritis, inflammatory bowel disease, and cancers [1,2]. The role of ATX in tissue repair is hijacked in cancers, which are likened to wounds that do not heal [4,5,6]. Chronic inflammation and decreased acquired immune responses are “hallmarks” of cancer [6,7]. Chronic LPA signaling enables cancer cells to evade the immune system [1]. LPA also stimulates VEGF production, which increases the angiogenesis needed for tumor growth.

ATX is secreted directly by melanoma, glioblastoma, and thyroid cancer cells [1]. By contrast, human and mouse breast cancer cells produce very little ATX. We earlier proposed a model for understanding breast cancer development in which inflammatory cytokines produced by tumors increase ATX secretion by surrounding breast adipocytes [1]. This amplifies the inflammatory cycle and promotes the accumulation of inflammatory macrophages [1]. Significantly, about 40% of ATX in mice is produced by adipocytes [8,9]. Our hypothesis was supported by the pharmacological inhibition of ATX, which decreased breast tumor growth and metastasis of 4T1 breast tumors in syngeneic BALB/c mice [1,10]. ATX inhibition also decreased the concentrations of multiple inflammatory cytokines and leukocyte infiltration in the adipose tissue [1,11]. Bi-directional signaling between breast tumors and surrounding adipose tissue through the ATX-LPA-inflammatory cycle has been confirmed [12,13]. The proposed role of adipose tissue in producing the ATX that drives breast cancer progression [1] could also provide an explanation for the association of an increased incidence of breast cancer with obesity [14,15].

We decided to test directly if breast adipocytes provide the ATX that drives breast tumor growth by using mice with an adipocyte-specific knockout of ATX. This did not affect the growth of syngeneic E0771 breast tumors in our orthotopic model with C57BL/6 or breast tumor growth and metastasis in the spontaneous MMTV-PyMT model. However, the inhibition of ATX by IOA-289 still decreased tumor growth from E0771 breast cancer cells, indicating that there is another source(s) of ATX that is driving tumor growth. We now propose that the ATX derived from tumor-associated fibroblasts, endothelial cells, and leukocytes provides the LPA that promotes breast tumor growth by changing the microenvironment of these breast tumors. This is supported by the effects of IOA-289 in increasing the numbers of CD8α^+^-T-cells in the tumors, decreasing the concentrations of CXCL10, CCL2, and CXCL9 in the plasma and LIF, TGFβ1, TGFβ2, and prolactin in the tumors. We conclude that inhibiting ATX activity from non-cancer cells in the breast tumor and the cross-talk involving LPA with breast cancer cells provides an effective strategy for reducing tumor growth and metastasis and for increasing the effectiveness of chemotherapy and radiotherapy.

## 2. Materials and Methods

### 2.1. Cell Lines and Reagents

The mouse breast cancer cell line E0771 was obtained from ATCC (Manassas, VA, USA) and cultured in DMEM (Dulbecco’s Modified Eagle’s Medium) supplemented with 10% FBS. Mycoplasma infection was excluded by a PCR test of the culture media using a mycoplasma detection kit (G238, Applied Biological Materials Inc, Richmond, BC, Canada). IOA-289 was supplied by iOnctura (Genève, Switzerland). Rabbit anti-CD45 (70257), rabbit anti-F4/80 (70076), rabbit anti-CD8α (98941), rabbit anti-CD206 (24595), rabbit anti-FoxP3 (12653), and rabbit anti-αSMA (19245) antibodies were from Cell Signaling Technology (Danvers, MA, USA), and Rabbit anti-CD31 antibody (ab182981) was from Abcam Inc. (Toronto, ON, Canada).

### 2.2. Establishment of Adipocyte-Specific ATX Knockout Mice as a Breast Cancer Model with IOA-289 Treatment

C57BL/6J-ENPP2^fl/fl^Adipoq-Cre^+^ and C57BL/6J-ENPP2^fl/fl^ mice were used in this study, and they were derived as described previously [16]. Briefly, the Cre recombinase gene in Cre^+^ mice is under control of the adiponectin promoter and therefore is specifically expressed in adipocytes. Male ENPP2^fl/fl^Adipoq-Cre^+^ mice were mated with female ENPP2^fl/fl^ mice, and the offspring were genotyped using a One-4-all Genomic DNA Miniprep kit (BS88505, Bio Basic Inc. Markham, ON, Canada).Primers for Cre are Forward: 5′-ACCTGAAGATGTTCGCGATT 3′ and Reverse: 5′ CGGCATCAACTGTTTCTTTT-3′; primers to distinguish ENPP2^fl/fl^ (290 bp product) and wild type ENPP2 (170 bp product) are forward: 5′-TGCTTGAAGTGTGTGTGCAC-3′ and reverse: 5′ CGGCATCAACTGTTTCTTTT-3′. Female mice with the genotype ENPP2^fl/fl^Adipoq-Cre^+^ were selected as adipocyte ATX knockout. Female offspring from ENPP2^fl/fl^ mice were used as controls. 

Syngeneic orthotopic mouse breast cancer models were established by inoculating 1.0 × 10^6^ E0771 breast cancer cells in PBS containing 50% Matrigel into the forth left mammary fat pad of female C57BL/6J-ENPP2^fl/fl^Adipoq-Cre^+^ (KO) and C57BL/6J-ENPP2^fl/fl^ (control) mice. Female mice were taken from our colony at 11–15 weeks of age for these experiments. The mice were housed under 12 h light/dark cycles with free access to water and standard mouse chow containing ~13% of calories (PicoLab Laboratory Rodent Diet 5LOD). Tumor growth was monitored by two orthogonal caliper measurements, and tumor volume was estimated using the equation width^2^ × length/2. IOA-289 was ground into a fine powder in a mortar and suspended at 20 mg/mL in 0.5% methyl cellulose (Cat.182312500, Acros Organics, Morris Plains, NJ, USA). Mice were gavaged orally with 100 mg/kg IOA-289 or vehicle twice per day, because ATX activity was recovered by ~88% over the 12 h between doses (Suppl. Figure S2C). This treatment began at Day 3 after the injection of cancer cells and was continued until Day 16 when the experiment was terminated. Plasma samples were collected within 3–5 h after the final dose of IOA-289. All procedures were performed in accordance with the Canadian Council of Animal Care as approved by the University of Alberta Animal Welfare Committee.

### 2.3. Establishment of Hybrid PyMT/Adipocyte ATX Knockout Mice and Monitoring Spontaneous Breast Tumor Development and Metastasis

To investigate the effect of adipocyte ATX knockout on initiation of breast tumor development, we crossed the C57BL/6-MMTV-PyMT mice, which develop spontaneous breast tumors, with C57BL/6J-ENPP2^fl/fl^Adipoq-Cre^+^ mice to generate adipocyte ATX knockout mice carrying PyMT. We first crossed male MMTV-PyMT mice with female C57BL/6J-ENPP2^fl/−^ Adipoq-Cre^+^ mice. Then, we selected F1 male ENPP2^fl/−^ mice that carry both PyMT and Cre and crossed them with female C57BL/6J-ENPP2^fl/−^ Adipoq-Cre^+^ mice again. We repeated the same breeding strategy by crossing F2 male ENPP2^fl/−^ mice that carry both PyMT and Cre with female C57BL/6J-ENPP2^fl/−^ Adipoq-Cre^+^ mice. Hybrid C57BL/6J-ENPP2^fl/fl^ Adipoq-Cre^+^-PyMT mice can be continuously generated, and the females were collected for monitoring the spontaneous breast tumor development. Primers for detect PyMT (556 bp product) are forward: 5′-GGA AGC AAG TAC TTC ACA AGG G-3′ and reverse: 5′-GGA AAG TCA CTA GGA GCA GGG-3′.

Female C57BL/6J-ENPP2^fl/fl^Adipoq-Cre^+^-PyMT mice were checked for spontaneous breast tumor twice a week from the age of 8-weeks. The time of finding palpable tumors was recorded, and the diameters of the tumors were monitored twice a week until the diameters reached 1 cm. The lungs were separated and perfused with 1 mL formalin through the trachea using a 1 mL syringe and stored in formalin. The paraffin embedding and sectioning for each lung are done in the same way that allows the lobes of both sides are presented on the slides. The lung micro-metastases were counted under a light microscope [17].

### 2.4. ATX Activity Measurement

ATX activity in mouse plasma was measured by determining choline release from LPC essentially as reported previously when we tested the pharmacokinetics of ONO-8430506 [10] and GLPG1690 [18] as ATX inhibitors. Briefly, 17 μL of plasma or 17 μL of buffer A (100 mM Tris-HCl, pH 9.0; 500 mM NaCl; 5 mM MgCl_2_; and 0.05% *v*/*v* Triton X-100) with 20% DMSO or 20% DMSO containing 8 mM IOA-289 was mixed with 8 μL of buffer A and preincubated at 37 °C for 30 min. Samples were then mixed with 25 μL of 600 µM C14:0-LPC in buffer A and incubated for a further 2 h at 37 °C. Samples (20 μL) of the first incubation mixtures were pipetted in duplicate into a 96-well plate and mixed with 90 μL/well of buffer C [88.3 μL Buffer B (100 mM Tris-HCl, pH 8.5, and 5 mM CaCl_2_), 0.58 μL of 10 mM Amplex Red, 0.12 μL of 1000 U/mL horseradish peroxidase, 1 μL of 50 U/mL choline oxidase]. Fluorescence was measured at Excitation 544 nm/Emission 590 nm, and choline concentrations were calculated from a standard curve. The samples containing the excess of IOA-289 in the assay served as a blank control to determine ATX-dependent choline formation and account for any free choline in the plasma.

It should be noted that the final LPC concentration in the first incubation is 300 μM, which is similar to the LPC concentration in blood. We normally use a saturating concentration of 4 mM LPC for the measurement of ATX activity, but this would favor the competitive displacement of IOA-289 from ATX and decrease the apparent inhibition from the IOA-289 in the plasma. Even so, some competition will occur at 300 µM LPC, and the level of inhibition reported should be considered as a minimum value.

### 2.5. Effects of Feeding a High fat Diet on ATX Activity and tumor Growth

We were aware that feeding a diet containing ~45% [8] or ~60% of calories from fat [16] was reported to increase ATX secretion from adipose tissue. Therefore, we thought that feeding mice with a high fat diet rather than using regular chow might provide a more appropriate model for studying breast cancer in women who typically consume a relatively high percentage of calories from fat. We, therefore, tested the effects of a nutritionally balanced diet containing 60% of calories from fat. This was achieved by adding 200 g fat consisting of 31 g Mazola Vegetable Oil, 167 g lard and 2 g of Arasco Oil (as a source of docosahexaenoic and arachidonic acids) to 800 g of a TD.84172 Basal mix diet from Harlan Laboratories (Madison, WI, USA). C57BL/6 mice were then fed ad libitum on this diet for 20 days prior to the injection of cancer cells and compared to mice fed on the normal laboratory chow (PicoLab Laboratory Rodent Diet 5LOD, LabDiet, Leduc, AB, Canada), which contained ~13% of calories as fat.

Feeding the high fat diet that was chosen for this work did not significantly alter the body weight, fat mass, or plasma ATX activity compared to mice fed on the normal chow (Suppl. Figure S1A–C). There were also no significant differences in the volumes or weights of the breast tumors with the limited numbers of mice studied (Suppl. Figure S1D,E). As a consequence of these results and the lack of an increased ATX activity on the high fat diet, we decided to use the regular diet for our studies on the effects of the KO of ATX in adipocytes on breast tumor growth.

### 2.6. Bioinformatics Analysis

Single-cell RNA sequencing data from two human breast tumor samples (GSM5354529 and GSM5354531) were downloaded from Gene Expression Omnibus database (https://www.ncbi.nlm.nih.gov/geo/query/acc.cgi?acc=GSE176078, accessed on 28 April 2023) and analyzed by the R-package Seurat. Cells with low (<200) and high (>7500) reads or >20% of mitochondrial reads were excluded from analysis. Data of the qualified cells were normalized, and non-linear dimensional reduction was performed using t-SNE. Cell cluster annotations were performed using EPCAM, KRT19, and PROM1 as epithelial (cancer) cell markers; PECAM1 and VWF as endothelial cell markers; COL1A1, COL1A2, and DCN as fibroblast cell markers; ACTA2, PDGFRB, and MCAM as perivascular-like cell markers; CD68, CD163, CSF1R, MRC1, TPSAB1, MS4A2, and CD1C as myeloid cell markers; CD79A, CD79B, and MS4A1 as B cell markers; CD3D, CD3E, CD8A, and CD4 as T cell markers.

Human breast tumor bulk RNA sequencing data of Metabric (2509 samples, https://www.cbioportal.org/study/summary?id=brca_metabric) and TCGA (1108 samples, https://www.cbioportal.org/study/summary?id=brca_tcga) were obtained from cBioPortal (https://www.cbioportal.org/, accessed on 20 April 2023). Multiple Pearson correlation analysis was performed by R. Genes significantly correlated with ATX (*ENPP2*) expression (Pearson correlation coefficient > 0.3, *p* < 0.05) were chosen for online analysis of gene ontology (GO) and Kyoto Encyclopedia of Genes and Genomes (KEGG) pathway (https://david.ncifcrf.gov/, accessed on 28 April 2023) to determine the biological process (BP), cellular component (CC), molecular function (MF), and pathways correlated with *ENPP2* expression. Gene set variation analysis (GSVA) was performed by R-package GSVA with default parameters. The GO gene sets were downloaded from the Gene Set Enrichment Analysis database (https://www.gsea-msigdb.org/, accessed on 28 April 2023). The correlation between *ENPP2* expression and the gene sets with biological terms was determined by Pearson correlation analysis.

### 2.7. Multiplex Protein Measurement

Cytokines, chemokines (31-ples, MD31) and growth factors (16-plex, MDAG16), matrix metalloproteases [MMPs (5-plex, MDMMP)] in plasma or excised tumor tissue were measured using multiplexing laser bead assay by Eve Technologies (Calgary, AB, Canada) as reported previously [11]. Sample preparation was performed following the instructions on the website of Eve Technologies (https://www.evetechnologies.com/sample-preparation-guide/, accessed on 28 April 2023).

### 2.8. Hydroxyproline Measurement

Hydroxyproline concentration in excised tumor tissue was determined with a hydroxyproline microplate assay kit (CAK1067) from Cohesion Bioscience (London, UK). Sample preparation and measurement were performed following the manual of the kit.

### 2.9. Quantitative PCR

Extraction of mRNA was performed using the EZ-10 DNAaway RNA miniprep kit (BS88136, Bio Basic Inc. Markham, ON, Canada) followed by reverse transcription using the All-In-One 5X RT MasterMix (G490, Applied Biological Materials Inc. Richmond, BC, Canada). Quantitative PCR was performed using BlasTaq™ 2X qPCR MasterMix (G892, Applied Biological Materials Inc. Richmond, BC, Canada). The primers for ATX are forward: 5′CATTTATTGGTGGAACGCAGA3′ and reverse: 5′CTACAAAAACAGTCTGCATGC3′. Cyclophilin A was used as an internal control. The primers for cyclophilin A are forward: 5′CACCGTGTTCTTCGACATCAC3′ and reverse: 5′CCAGTGCTCAGAGCTCGAAAG3′.

### 2.10. Immunohistochemistry

Immunohistochemistry was performed on 5 μm paraffin-embedded tumor sections using the Rabbit specific HRP/DAB (ABC) Detection IHC Kit (ab64261, Abcam, Toronto, ON, Canada) according to the manufacturer’s instructions. Antigen retrieval was performed by microwaving hydrated slides in a pressure cooker for 20 min in citrate buffer (10 mM citric acid, 0.05% Tween 20, pH 6.0). Images were acquired using a Zeiss Axioskop 2 imaging system (Carl Zeiss Canada, Toronto, ON, Canada). Five images were analyzed to calculate the average value for each slide.

### 2.11. Tumor Digestion and Cell Sorting

E0771 cells stably expressing GFP were injected into C57BL/6 mice to create tumors as described above. To obtain single cells, E0771-GFP breast tumors were minced with scissors and digested with 1 mg/mL type IV collagenase, 0.1 mg/mL type V hyaluronidase, and 20 U/mL type IV DNase in calcium-free PBS at 37 °C for 3 h. Cell suspension was washed with calcium-free PBS for three times and stained with anti-CD45 PE-Texas Red (MCD4517, ThermoFisher Scientific, Waltham, MA, USA), anti-fibroblast activation protein (FAP) Alexa Fluor 594 (983802, Bio-Techne, Toronto, ON, Canada) and Live/Dead Fixable Near IR780 (L34994, ThermoFisher Scientific, Waltham, MA, USA). GFP^+^ E0771 breast cancer cells, CD45^+^ leukocytes, and FAP^+^ fibroblasts were separated and collected with Sony MA900 cell sorter.

### 2.12. Statistical Analysis

Results are expressed as means ± SEM and were analyzed by student *t*-test or ANOVA followed by Tukey’s test using GraphPad Prism 9 (La Jolla, CA, USA). *p* < 0.05 was considered statistically significant.

## 3. Results

### 3.1. Breast Tumor Growth Was Suppressed by Inhibiting ATX with IOA-289 but Not by ATX Knockout in Adipocytes

**Figure 1:**
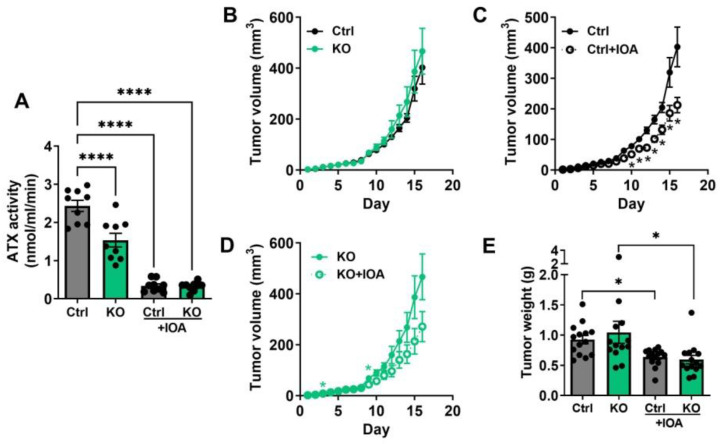
(**A**) Plasma ATX activity of C57BL/6J-ENPP2^fl/fl^ (Ctrl) and C57BL/6J-ENPP2^fl/fl^Adipoq-Cre^+^ (KO) mice with or without IOA-289 (IOA) treatment (gavaged twice a day at 100 mg/kg body weight for 5 days). Plasma was collected between 1–3 h after the final dose of IOA-289. n = 9 per group. (**B**–**D**) Daily tumor volume and (**E**) tumor weight of Ctrl and KO mice with or without IOA treatment (gavaged twice a day at 100 mg/kg body weight). n = 14 per group, * *p* < 0.05, **** *p* < 0.0001.

We established a mouse model for this study by breeding C57BL/6J-ENPP2^fl/fl^Adipoq-Cre^+^ mice carrying adipocyte-specific ATX knockout. These mice showed a ~37% decrease in plasma ATX activity compared with the C57BL/6J-ENPP2^fl/fl^ control mice (Figure 1A). This reflects the contribution of adipocytes to total ATX production as expected [8]. The decrease in ATX activity in abdominal adipose tissue was ~67% (Suppl. Figure S2A). The activity of ATX in adipose tissue also correlated strongly with plasma ATX activity (Suppl. Figure S2B). IOA-289 treatment of the mice suppressed plasma ATX activity by a minimum estimate of 86% in the control mice and 79% in the adipocyte-ATX KO mice (Figure 1A). These results establish the validity of the ATX knockouts in adipocytes and the efficacy of IOA-289 in blocking ATX activity in vivo.

We next tested the effect of the ATX KO on tumor growth using E0771 breast cancer cells, which are syngeneic with the C57BL/6 strain on which the KO was created. Surprisingly, tumor growth of orthotopically implanted E0771 cells in adipocyte-ATX KO mice showed no significant difference relative to the control mice when measured by daily tumor volume or terminal tumor weight at Day 16 after injection of the cancer cells (Figure 1B,E). To test the effects of blocking all ATX activity, mice were treated with IOA-289, which is a potent selective ATX inhibitor that is effective in animals [19], by gavage twice a day at 100 mg/kg body weight starting from day 3 after injection of cancer cells. The last dose was given on day 16 before sacrificing the mice 3–5 h later. IOA-289 was well tolerated as assessed by no significant effects on weight gain over the 13 days of treatment (Suppl. Figure S2D). IOA-289 did decrease tumor volume (Figure 1C,D) and the more accurately measured tumor weight (Figure 1E), by ~31% and 43%, respectively, in the control mice and mice with the adipocyte-ATX KO. These results indicated that a source of ATX other than from adipocytes was responsible for promoting tumor growth.

**Figure 2:**
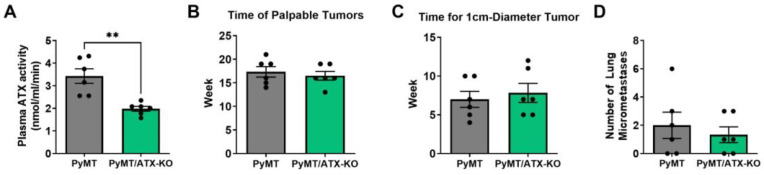
(**A**) Plasma ATX activity of MMTV-PyMT (PyMT) and MMTV-PyMT-ENPP2^fl/fl^Adipoq-Cre^+^ (PyMT/ATX-KO) mice. (**B**) Ages of palpable tumors can be observed in PyMT and PyMT/ATX-KO mice. (**C**) Time of formation of 1 cm-diameter tumors since being palpable in PyMT and PyMT-ATX-KO mice. (**D**) Numbers of micro-metastases in lung sections of PyMT and PyMT-ATX-KO mice. ** *p* < 0.01.

We also verified the effect of KO for ATX in adipocytes by crossing these mice with MMTV-PyMT mice that are also on the C57BL/6 background. Female mice develop spontaneous breast tumors after about 17 weeks of age. KO of ATX in adipocytes decreased the plasma activity of ATX by ~40% (Figure 2A), but again, KO of ATX in adipocytes did not affect tumor growth as indicated by the age at which palpable tumors were first detected, or the time for a tumor to reach 1 cm in diameter (Figure 2B,C). KO of ATX also did not appear to change the number of lung metastases that were detected (Figure 2D).

These combined results demonstrate that MMTV-PyMT breast tumor growth does not depend on ATX produced by adipocytes that surround the tumor, but some other source of ATX is involved.

### 3.2. Identification of Cells in E0771 and Human Breast Tumors That Can Synthesize ATX and the Functions of ATX in the Human Breast Tumor Microenvironment

**Figure 3:**
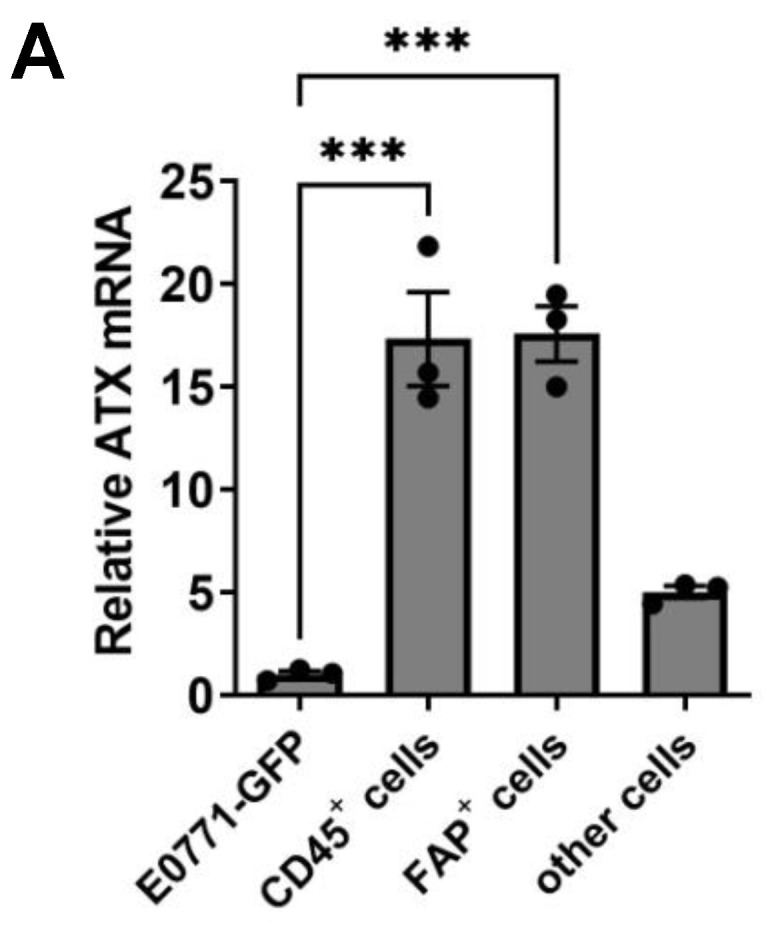

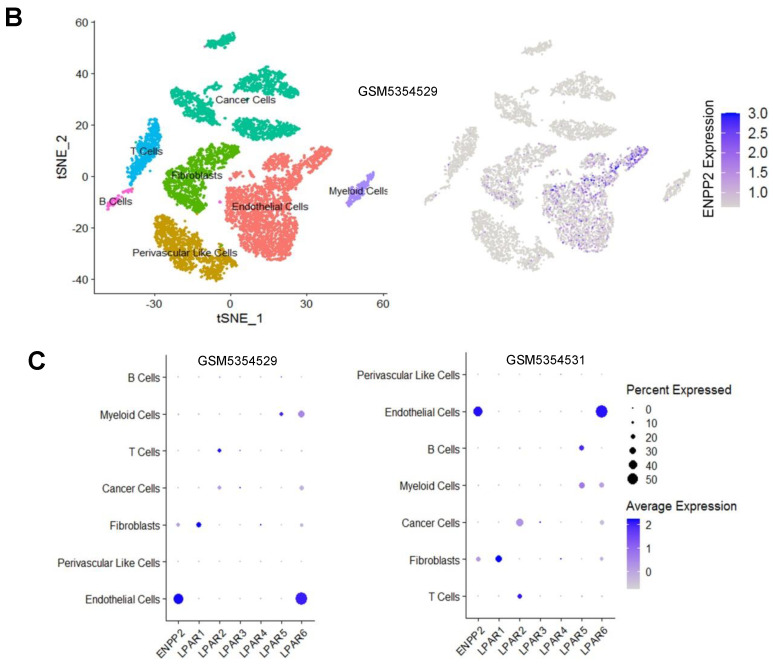
(**A**) ATX (*ENPP2*) mRNA levels of different cells in breast tumors developed from E0771 cells expressing GFP in wild-type C57BL/6 mice. GFP^+^ E0771 breast cancer cells, CD45^+^ leukocytes, and FAP^+^ fibroblasts were separated with FACS. (**B**) Cell clusters and *ENPP2* expression in human breast tumor (GSM5354529). (**C**) Expression of *ENPP2* and LPA receptor 1–6 in different types of cells of two human breast tumors (GSM5354529 and GSM5354531). *** *p* < 0.001.

To investigate which tumor cells are likely to secrete ATX, we implanted breast tumors in mice using E0771 cells that expressed green fluorescent protein (GFP). The tumors were then digested into individual cells, and FACS was used to isolate GFP-expressing cancer cells, CD45^+^ leukocytes, fibroblast activation protein (FAP)^+^ fibroblasts, and other cells. Measurements of ATX mRNA show that the majority of ATX is expressed by fibroblasts and leukocytes (Figure 3A).

To determine the source of ATX in human breast tumors, we analyzed single-cell RNA sequencing data of two human breast tumors (GSM5354529 and GSM5354531). Similarly, ATX in human breast tumors also showed a tumor stroma-derived manner. Different from the mouse breast cancer model, the majority of ATX in human breast tumors was expressed by endothelial cells and a small portion of fibroblasts (Figure 3B,C), whereas LPA receptors in the tumors were widely expressed by cancer cells, fibroblasts, T cells, myeloid cells, and endothelial cells (Figure 3C).

**Figure 4:**
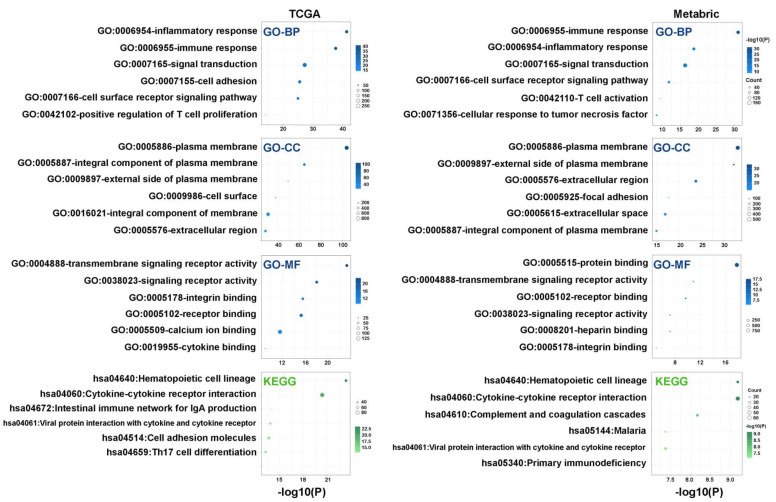
GO and KEGG pathway enrichment analysis for *ENPP2* correlated genes from TCGA and Metabric data sets. Six biological processes (BP), cellular components (CC), molecular functions (MF), and KEGG pathways where these genes are most significantly enriched were shown as bubble charts.

We then performed gene ontology (GO) enrichment analysis to identify ATX (*ENPP2*) related biological functions in human breast tumors. Genes from TCGA and Metabric data sets that are significantly correlated with *ENPP2* expression (Pearson correlation r > 0.3 and *p* < 0.05) were analyzed, and six biological processes (BP), components (CC), molecular functions (MF), and KEGG pathways in which these genes are most significantly enriched were showed in Figure 4. Both of the gene sets from TCGA and Metabric showed significant enrichment in BPs including “inflammatory response”, “immune response”, “signal transduction”, and “cell surface receptor signaling pathway”; in CCs including “plasma membrane”, “integral component of plasma membrane”, “external side of plasma membrane”, and “extracellular region”; in MFs including “transmembrane signaling receptor activity”, “signaling receptor activity”, “integrin binding”, and “receptor binding”; in KEGG pathways including “hematopoietic cell lineage”, “cytokine-cytokine receptor interaction”, and “viral protein interaction with cytokine and cytokine receptor”. These results indicated that ATX functions as a paracrine regulator of cancer cells and is involved in cytokine signaling, which regulates inflammatory and immune responses in the breast tumor microenvironment.

**Figure 5:**
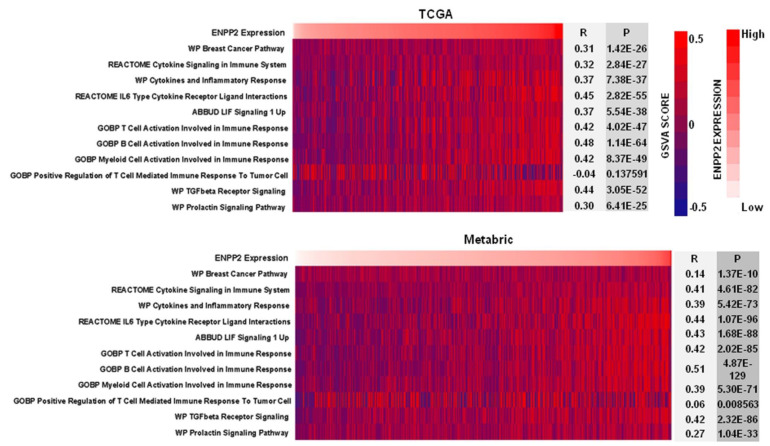
Gene set variation analysis (GSVA) for *ENPP2* in TCGA and Metabric data sets. GSVA scores for gene sets with different gene ontology terms were calculated and expressed as heat maps. Pearson correlation analysis was performed to determine the correlation between *ENPP2* expression and these gene ontology terms (biological functions) in human breast tumors.

To further clarify the role of ATX in cytokine signaling as well as inflammatory and immune responses in breast cancer, gene set variation analysis (GSVA) of *ENPP2* was performed for TCGA and Metabric data sets. The GSVA scores of each sample for different gene sets of interest were calculated using R-package GSVA, and the correlation between the GSVA scores and *ENPP2* expression of each sample was determined by Pearson correlation analysis. As expected, *ENPP2* showed significant correlations with biological functions of “cytokine signaling in immune system”, “cytokines and inflammatory response”, “T cell activation involved in immune response”, “B cell activation involved in immune response”, and “myeloid cell activation involved in immune response”. Especially, *ENPP2* was significantly correlated with “IL-6 cytokine receptor ligand interactions”, “LIF signaling 1 up”, “TGFβ receptor signaling” and “prolactin signaling pathway” in human breast tumors (Figure 5).

### 3.3. IOA-289-Induced Changes on Plasma Cytokines/Chemokines in Blood Plasma and Tumors

**Figure 6:**
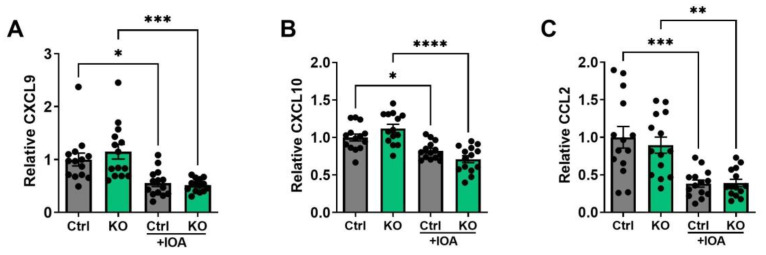
Plasma concentrations of CXCL9 (**A**), CXCL10 (**B**), and CCL2 (**C**) in C57BL/6J-ENPP2^fl/fl^ (Ctrl) and C57BL/6J-ENPP2^fl/fl^Adipoq-Cre^+^ (KO) mice with or without IOA-289 (IOA) treatment. The average value of the control group is used for normalization. n = 14 per group, * *p* < 0.05, ** *p* < 0.01, *** *p* < 0.001, **** *p* < 0.0001.

**Figure 7:**
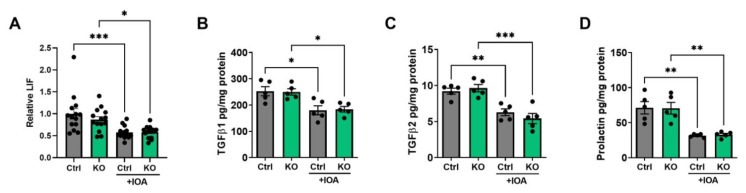
Tumor concentrations of LIF (**A**), TGFβ1 (**B**), TGFβ2 (**C**), and prolactin (**D**) in C57BL/6J-ENPP2^fl/fl^ (Ctrl) and C57BL/6J-ENPP2^fl/fl^Adipoq-Cre^+^ (KO) mice with or without IOA-289 (IOA) treatment. n = 14 per group for LIF, the average value of the control group was used for normalization. n = 5 per group for TGFβ1, TGFβ2, and prolactin. * *p* < 0.05, ** *p* < 0.01, *** *p* < 0.001.

**Figure 8:**
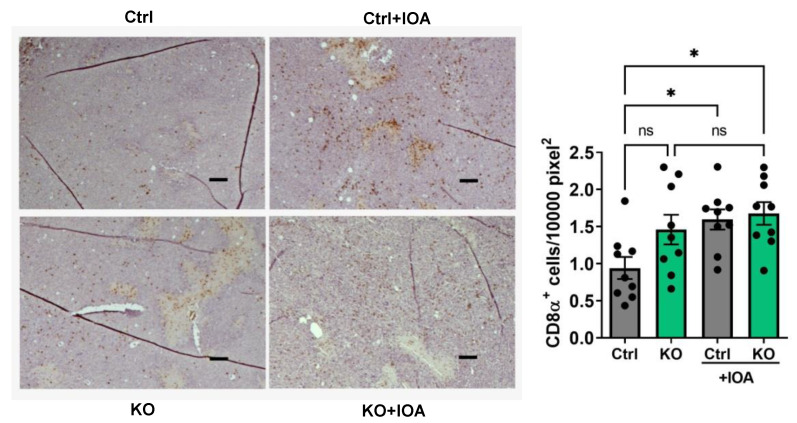
IHC staining and quantification of CD8α^+^ T-cells in the tumors of C57BL/6J-ENPP2^fl/fl^ (Ctrl) and C57BL/6J-ENPP2^fl/fl^Adipoq-Cre^+^ (KO) mice with or without IOA-289 (IOA) treatment. Scale bar = 100 μm. n = 9, * *p* < 0.05, not significant: *p* > 0.05.

Based on the evidence obtained from the bioinformatics analysis, we then determined possible LPA-induced changes in the concentrations of cytokines, chemokines, growth factors, and matrix metalloproteinase (MMPs) in the blood plasma and tumors. Multiplex protein measurements showed that IOA-289 treatment in wild-type mice and those with the adipocytes ATX KO significantly decreased concentrations of CXCL9, CXCL10, and CCL2 in plasma (Figure 6A–C). In the tumors, inhibition in ATX activity decreased the concentrations of LIF, TGFβ1, TGFβ2, and prolactin (Figure 7A–D). These results were consistent with the GSVA analysis in human breast tumors. Concentrations of other tested cytokines, growth factors, and five MMPs were not affected by IOA-289 (Suppl. Figures S3–S6). *ENPP2* expression in human breast tumors showed a positive correlation with leukocyte activation involved in immune response, which is probably associated with the chronic inflammation milieu in the tumor microenvironment. Inhibiting ATX by IOA-289 significantly increased the number of infiltrating CD8α^+^-T-cells in the wild-type mice (Figure 8). The numbers of CD8α+ T-cells in the tumors of the ATX-KO mice that were not treated with IOA-289 also appeared to be higher than the non-treated control mice, but this difference was not significant (*p* = 0.13) with the number of mice used. IOA-289 did not increase the infiltration of CD8α^+^ T-cells in the ATX-KO mice. Infiltration of CD45^+^ leukocytes, F4/80^+^ macrophages, CD206^+^ M2 subset of macrophages, and FOXP3^+^ regulatory T-cells in tumors were not affected by IOA-289 in this model (Suppl. Figure S7).

There were no significant effects of IOA-289 on blood vessels and myofibroblasts in the tumors as measured by IHC with CD31 and αSMA, respectively (Suppl. Figure S7), or hydroxyproline content of the tumors, as an indicator of fibrosis (Suppl. Figure S8).

## 4. Discussion

Our present work studied mice in which ATX was knocked out (KO) in adipocytes. This did not alter the growth of injected syngeneic E0771 breast tumors in this model, or the formation, growth, and metastasis of breast tumors in MMTV-PyMT mice where tumors develop spontaneously. These results disprove the hypothesis that adipocytes supply the majority of ATX that promotes breast tumor growth and metastasis. We then used the ATX inhibitor, IOA-289, which is now in Phase 1B trials for the treatment of pancreatic cancer [20], to test if it could attenuate breast tumor growth in the control mice or those with the KO of ATX in adipocytes. These experiments demonstrated that IOA-289 inhibited breast tumor growth in wild-type mice and also in mice with the KO of ATX in adipocytes. This indicates that there is a source of ATX other than adipocytes that drives tumor growth. This is most likely to be provided by fibroblasts and/or leukocytes within the E0771 tumors. The cells that produce the majority of ATX within breast tumors probably depend on the tumor model [21].

In human breast tumors, the ATX is also produced by stromal cells, but endothelial cells are the major source of ATX. A small portion of fibroblasts also express ATX. In contrast to the restricted source of ATX, LPA receptors are widely expressed by cancer cells, fibroblasts, leukocytes, and endothelial cells in human breast tumors. Inhibiting ATX activity in the E0771 mouse model not only affected LPA signaling in the cancer cells but also in stromal cells, thus changing the tumor microenvironment. Blocking ATX activity changed the phenotype of the mice by decreasing the plasma concentrations of CXCL9, CXCL10, and CCl2 and the tumor concentrations of LIF, TGFβ1, TGFβ2, and prolactin. These results are compatible with the results of GSVA analysis in human breast tumors and indicate that the ATX-LPA axis modulates LIF, TGFβ, and prolactin signaling that are involved in breast tumor development.

CCL2 (MCP-1) is a potent chemokine for monocytes, macrophages, and other immune cells. Blocking its activity decreases the growth and progression of tumors including breast tumors [22,23,24]. CXCL9 and CXCL10 through their receptor CXCR3 regulate immune cell migration, differentiation, and activation, leading to tumor suppression under some conditions [25]. However, CXCCL-9, CXCL10, and CXCR3 can have an opposite action in vivo where they increase tumor growth [26,27]. CXCL10 has also been proposed as a target for cancer therapy [28]. This appears to be the case in the present studies where IOA-289 decreased the plasma concentrations of CCL2, CXCL9, and CXCL10, together with its effect of inhibiting the growth of E0771 breast tumors.

The bioinformatics analysis in human breast tumors demonstrated that *ENPP2* is significantly correlated with cytokine signaling, leukocyte activation, inflammation, and immune response, suggesting that *ENPP2* is associated with the chronic inflammation milieu in the breast tumor microenvironment. Significantly, IOA-289 increased the concentration of infiltrating CD8^+^-T-cells in the E0771 breast tumors of wild-type C57BL/6 mice, and this could contribute to the net decrease in tumor growth. However, there was no significant effect of IOA-289 on the infiltration of CD8^+^-T-cells in the tumors of the ATX-KO mice. This could have been because ATX production from adipocytes decreases the accumulation of CD8^+^-T-cells in the tumors as well as ATX production from cells from the tumor. IOA-289 would have blocked the activity of ATX from all sources. More work is required to evaluate the relative roles of ATX derived from adipocytes and different tumor cells in the recruitment of CD8^+^-T-cells. Our studies complement recently published work showing that IOA-289 increased the infiltration of CD8^+^-T-cells into E0771 breast tumors in C57BL/6 mice and 4T1 breast tumors in BALB/c mice, and this was accompanied by decreased tumor growth [19]. These effects of ATX inhibition are also compatible with previous work in which ATX and LPA suppressed the infiltration of tumors with CD8^+^-T-cells [29,30].

IOA-289 can also modify the concentrations of cytokines/chemokines and growth factors in the tumors by decreasing LPA signaling, and this is relevant to net tumor growth. LIF is a member of the pro-inflammatory IL-6 family of cytokines and as such a potential contributor to tumor growth [31]. Targeting LIF and its receptor (LIFR/CD118) has been proposed as a potential treatment for solid tumors [32]. TGFβ signaling can have a tumor suppressor effect in early-stage cancers, but it is pro-metastatic in late-stage tumorigenesis [33]. TGF-β signaling regulates several events of tumor biology including cell cycle regulation, apoptosis, angiogenesis, immune suppression, migration, and invasion. This makes TGF-β signaling an attractive target for anti-cancer therapy, including breast cancer [34]. High exposure of breast tumors to prolactin has been associated with an increased risk of breast cancer, particularly estrogen receptor-positive cancers. Targeting downstream signaling from the prolactin receptor could offer further therapeutic targets [35]. It could, therefore, be significant that IOA-289 decreased the concentrations of LIF, TGF-β1/2, and prolactin in breast tumors, at least in the E0771 mouse model of breast cancer.

We needed to use C57BL/6 mice with syngeneic E0771 breast cancer cells or to cross-breed MMTV-PyMT with the adipocyte-specific ATX KO in this work because that was the background of the transgenic mice. The E0771 model of breast cancer does not result in metastasis, and it is not a very inflammatory model compared to the use of BALB/c mice with syngeneic 4T1 breast cancer cells. In the latter model, inhibition of ATX by ONO-8430506 decreased the concentrations of 20 inflammatory mediators and leukocytes in the inflamed adipose tissue adjacent to the breast tumor and the concentrations of G-CSF and TNFα in the plasma [11]. This was accompanied by decreased tumor growth and metastasis [10], especially when ONO-8430506 was combined with doxorubicin [36]. These studies illustrate that breast adipose tissue becomes inflamed by the presence of breast tumors in mouse models and that increased ATX expression is involved in the inflammatory milieu of the cancerous breast.

Despite this, the present work demonstrates that ATX produced by adipocytes is not driving breast tumor growth, but other sources of ATX such as tumor-associated fibroblasts and leukocytes in the E0771 model are probably much more important. This concept is compatible with the view that secreted ATX acts locally by binding to the surface of neighboring cells channeling LPA to its receptors [37,38]. In fact, chemokine-induced increases in ATX receptor expression, and binding of ATX to lymphocytes has been suggested as a mechanism for increased LPA action at sites of chronic inflammation [39]. Significantly, increased ATX activity is associated with increased atherosclerosis in mice [40], and deleting ATX expression in endothelial cells improves the outcomes of stroke [41]. Our results for the lack of effect of the adipocyte-specific KO for ATX on breast tumor growth do not support the proposition [1] that ATX produced by the increased mass of adipose tissue in obesity provides an explanation for an association with an increased incidence of breast cancer [14,15]. Despite this, increased ATX secretion from inflamed adipocytes could have other consequences, e.g., the recruitment of CD8^+^-T-cells and response of breast tumors to chemotherapy and radiotherapy. However, the ATX inhibitor, IOA-289, appears to have a more direct effect on E0771 tumor growth by targeting ATX secreted by cells such as fibroblasts and leukocytes within the tumor.

## Data Availability

Data is contained within the article.

## Supplementary Materials

The following supporting information can be downloaded at: https://www.mdpi.com/article/10.3390/cancers15112937/s1, Figure S1: Effects of high fat feeding; Figure S2: Correlation between adipose tissue and plasma; Figure S3: Cytokine concentrations in the plasma; Figure S4: Cytokine concentrations in the tumors; Figure S5: Concentrations of TGFβ3, EGF, FGF-2, and HGF in the tumors; Figure S6: Concentrations of MMPs in the tumors; Figure S7: Quantification of IHC staining for leukocytes. Figure S8: Concentrations of hydroxyproline in the tumors of C57BL/6J-ENPP2^fl/fl^ (Ctrl) and C57BL/6J-ENPP2^fl/fl^ Adipoq-Cre^+^ (KO) mice with or without IOA-289 (IOA) treatment. N = 9 per group, ns: *p* > 0.05.

## Abbreviations

ATX	autotaxin
IHC	immunocytochemistry
LPA	Lysophosphatidate, lysophosphatidic acid
LPC	Lysophosphatidylcholine
KO	knockout
MMP	matrix metalloproteinase
