# Supplementary material for: Autotaxin Inhibition with IOA-289 Decreases Breast Tumor Growth in Mice Whereas Knockout of Autotaxin in Adipocytes Does Not
Source: Cancers (Basel). 2023 May 26;15(11):2937. doi: 10.3390/cancers15112937 (PMC10251959; doi:10.3390/cancers15112937)
Supplement: Supplementary file 1 [file cancers-15-02937-s001.zip › cancers-2374581-supplementary.pptx]

## Slide 1
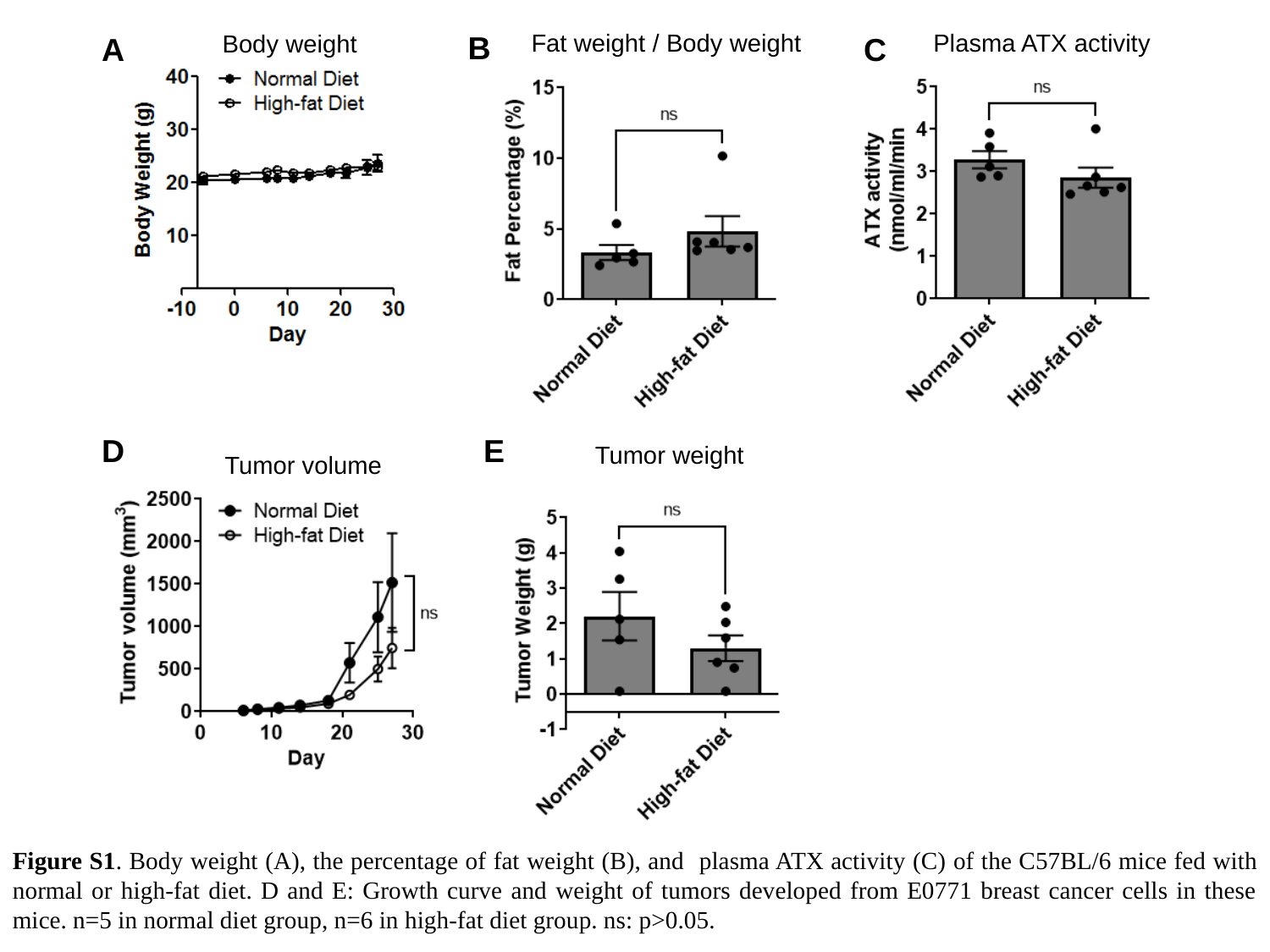

Fat weight / Body weight
Plasma ATX activity
Body weight
B
A
C
D
E
Tumor weight
Tumor volume
Figure S1. Body weight (A), the percentage of fat weight (B), and plasma ATX activity (C) of the C57BL/6 mice fed with normal or high-fat diet. D and E: Growth curve and weight of tumors developed from E0771 breast cancer cells in these mice. n=5 in normal diet group, n=6 in high-fat diet group. ns: p>0.05.

## Slide 2
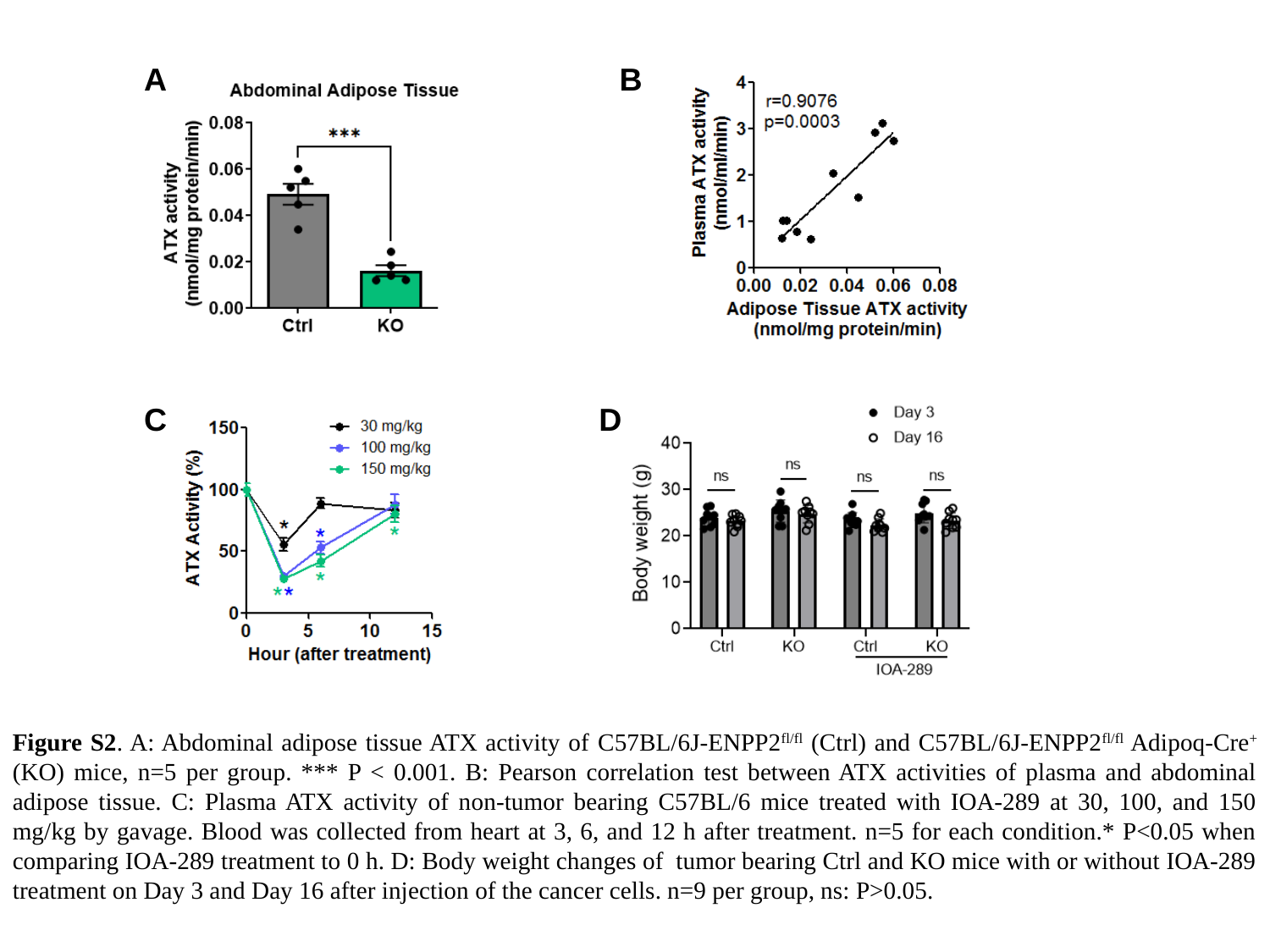

A
B
C
D
Figure S2. A: Abdominal adipose tissue ATX activity of C57BL/6J-ENPP2fl/fl (Ctrl) and C57BL/6J-ENPP2fl/fl Adipoq-Cre+ (KO) mice, n=5 per group. *** P < 0.001. B: Pearson correlation test between ATX activities of plasma and abdominal adipose tissue. C: Plasma ATX activity of non-tumor bearing C57BL/6 mice treated with IOA-289 at 30, 100, and 150 mg/kg by gavage. Blood was collected from heart at 3, 6, and 12 h after treatment. n=5 for each condition.* P<0.05 when comparing IOA-289 treatment to 0 h. D: Body weight changes of tumor bearing Ctrl and KO mice with or without IOA-289 treatment on Day 3 and Day 16 after injection of the cancer cells. n=9 per group, ns: P>0.05.

## Slide 3
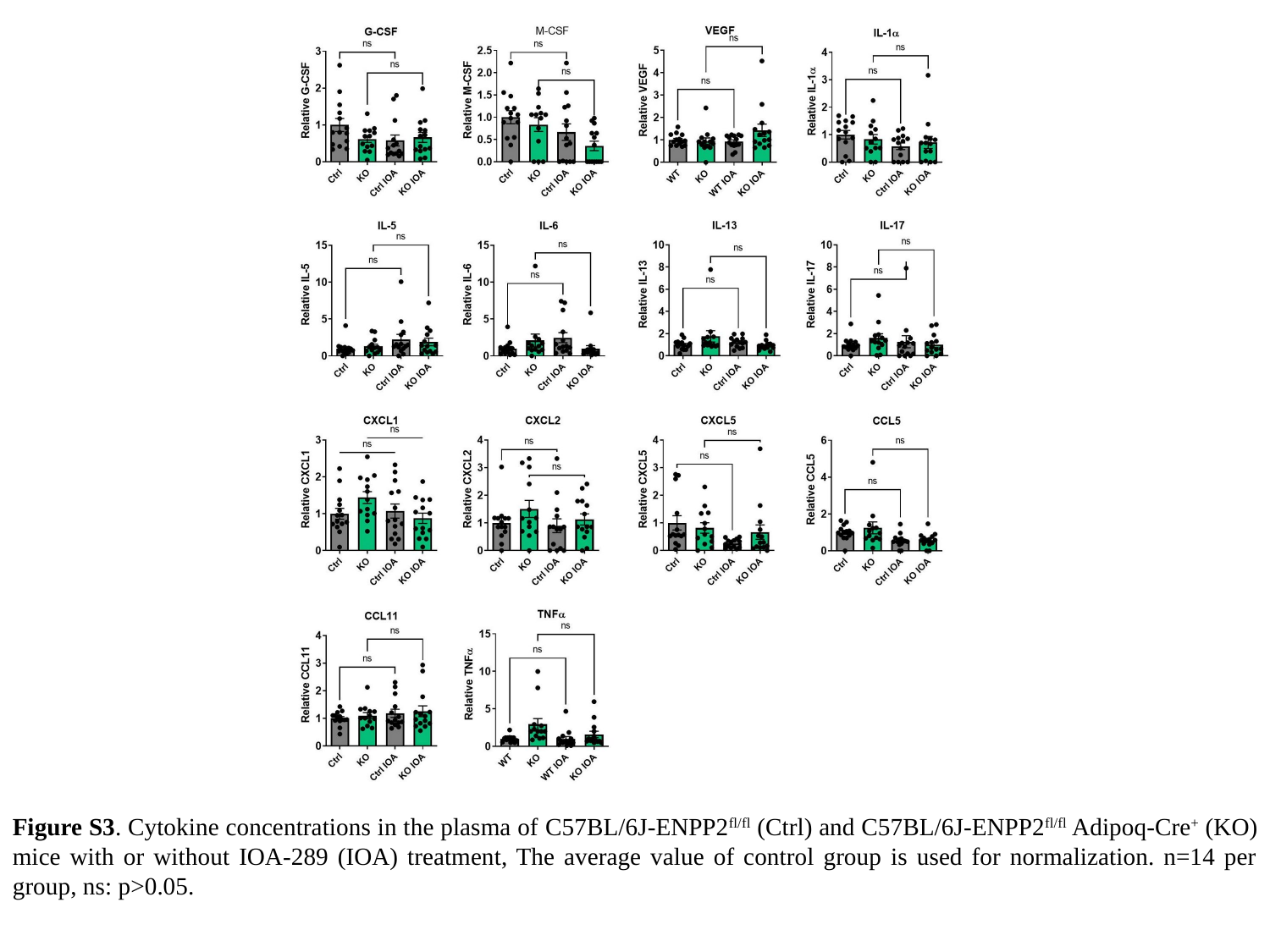

Figure S3. Cytokine concentrations in the plasma of C57BL/6J-ENPP2fl/fl (Ctrl) and C57BL/6J-ENPP2fl/fl Adipoq-Cre+ (KO) mice with or without IOA-289 (IOA) treatment, The average value of control group is used for normalization. n=14 per group, ns: p>0.05.

## Slide 4
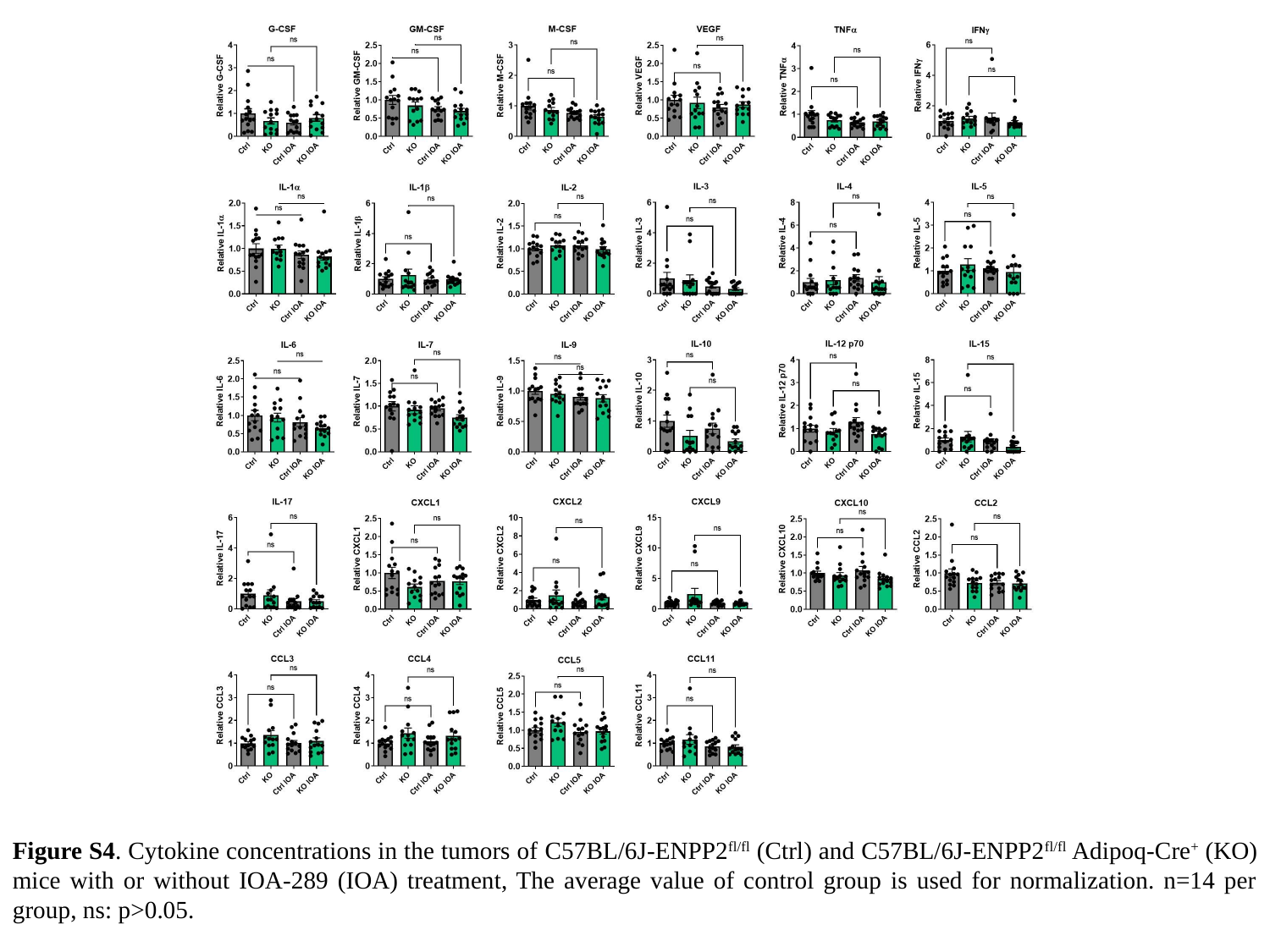

Figure S4. Cytokine concentrations in the tumors of C57BL/6J-ENPP2fl/fl (Ctrl) and C57BL/6J-ENPP2fl/fl Adipoq-Cre+ (KO) mice with or without IOA-289 (IOA) treatment, The average value of control group is used for normalization. n=14 per group, ns: p>0.05.

## Slide 5
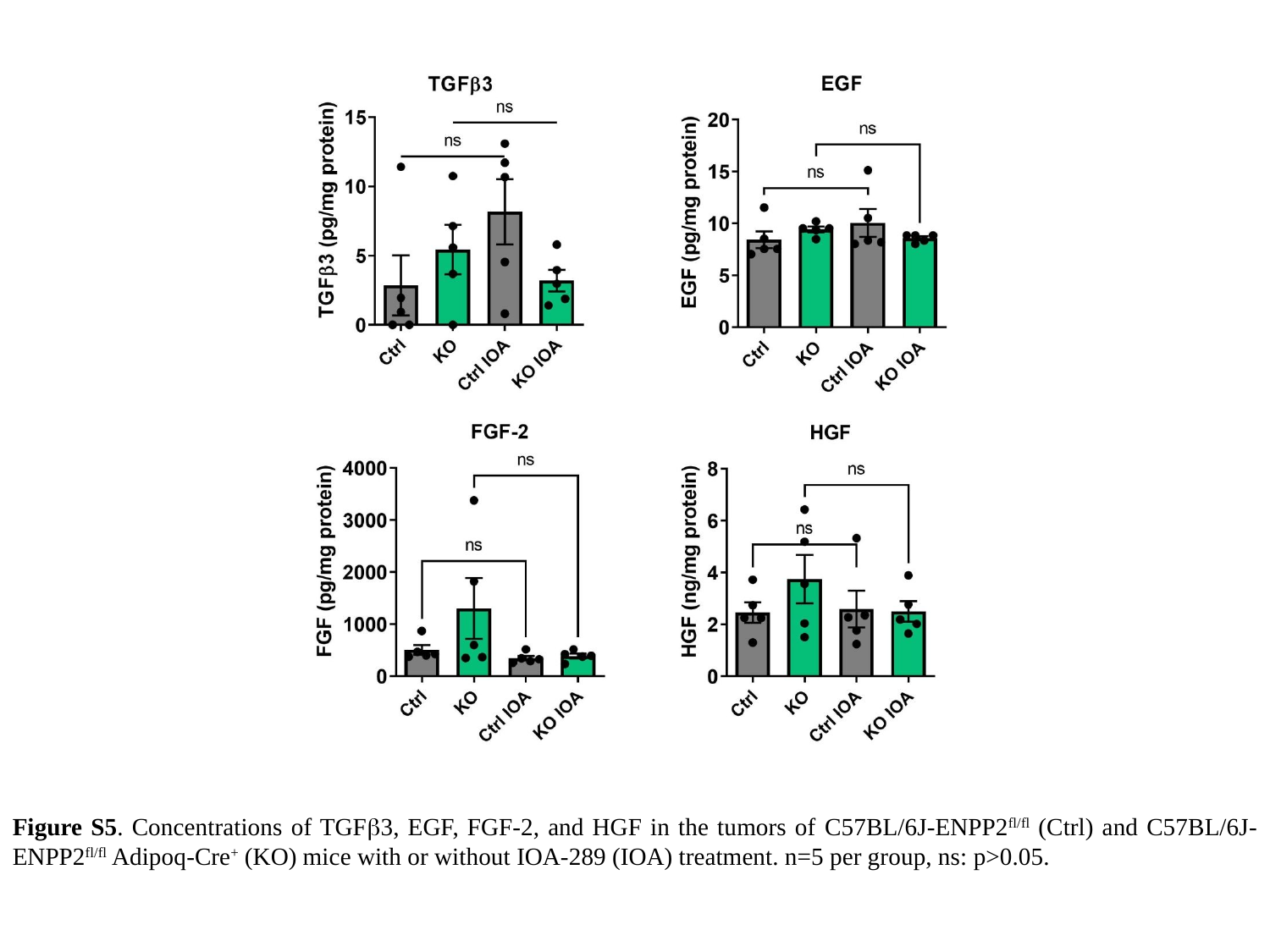

Figure S5. Concentrations of TGFb3, EGF, FGF-2, and HGF in the tumors of C57BL/6J-ENPP2fl/fl (Ctrl) and C57BL/6J-ENPP2fl/fl Adipoq-Cre+ (KO) mice with or without IOA-289 (IOA) treatment. n=5 per group, ns: p>0.05.

## Slide 6
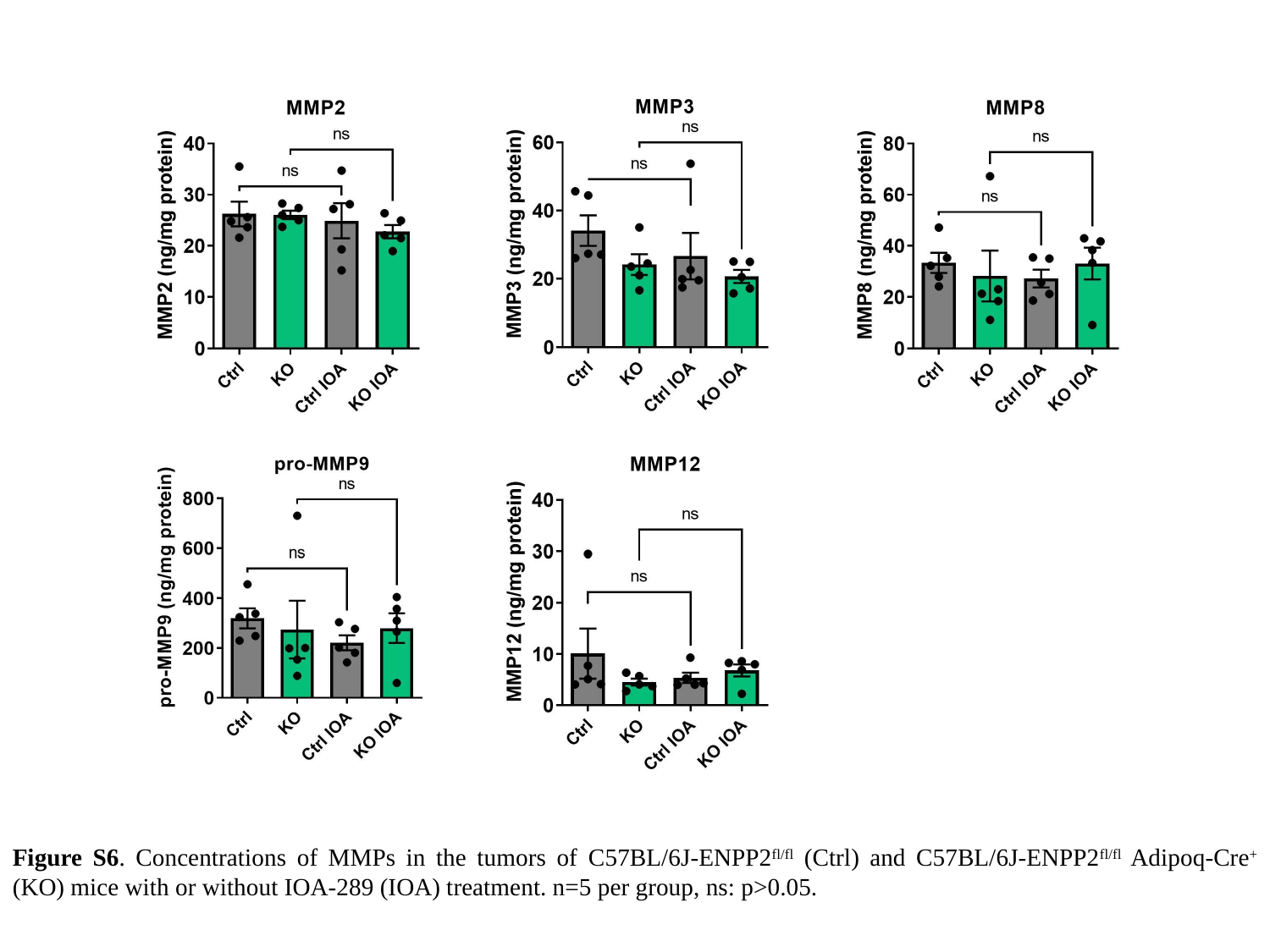

Figure S6. Concentrations of MMPs in the tumors of C57BL/6J-ENPP2fl/fl (Ctrl) and C57BL/6J-ENPP2fl/fl Adipoq-Cre+ (KO) mice with or without IOA-289 (IOA) treatment. n=5 per group, ns: p>0.05.

## Slide 7
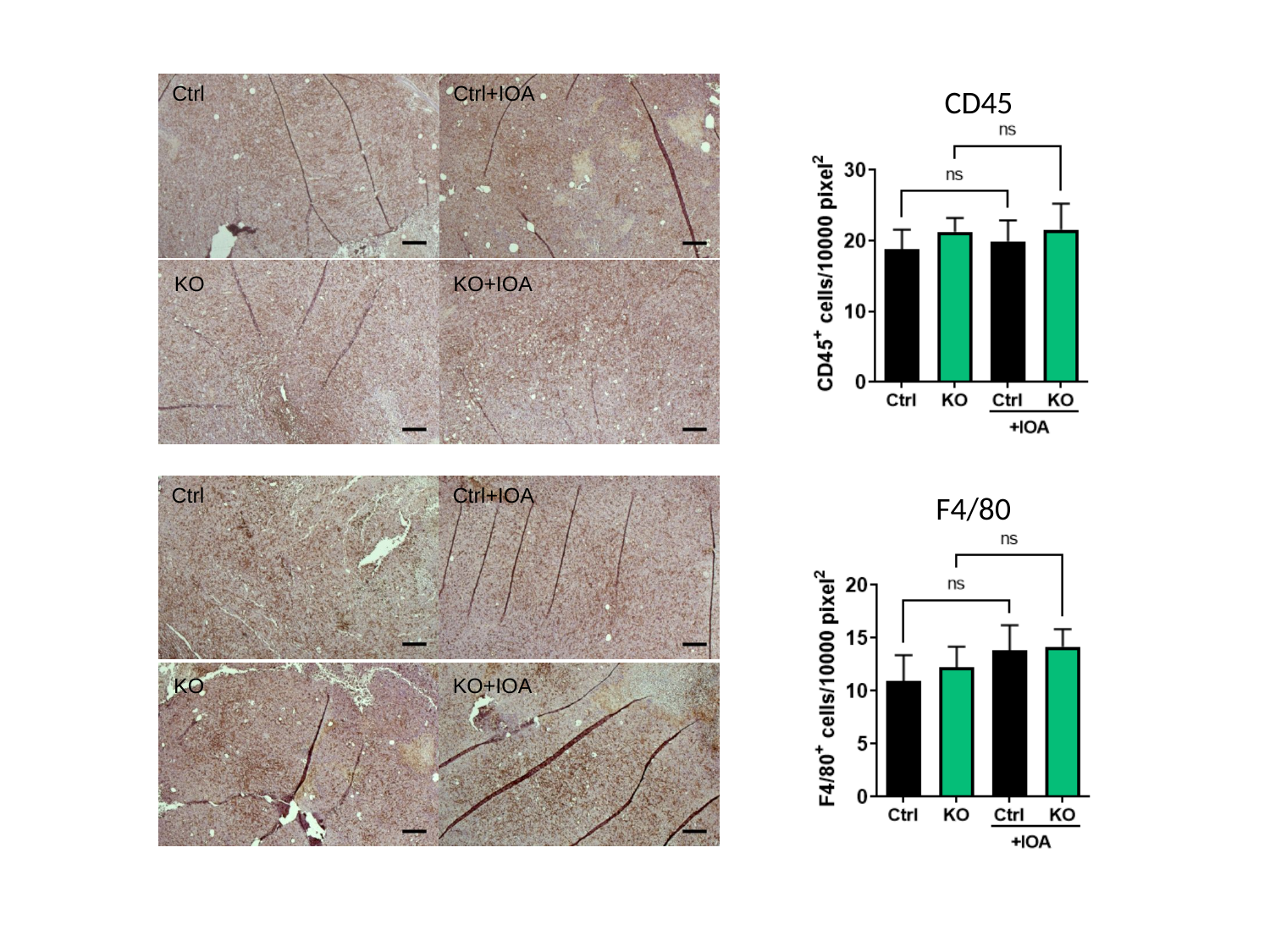

Ctrl
Ctrl+IOA
KO
KO+IOA
CD45
Ctrl
Ctrl+IOA
KO
KO+IOA
F4/80

## Slide 8
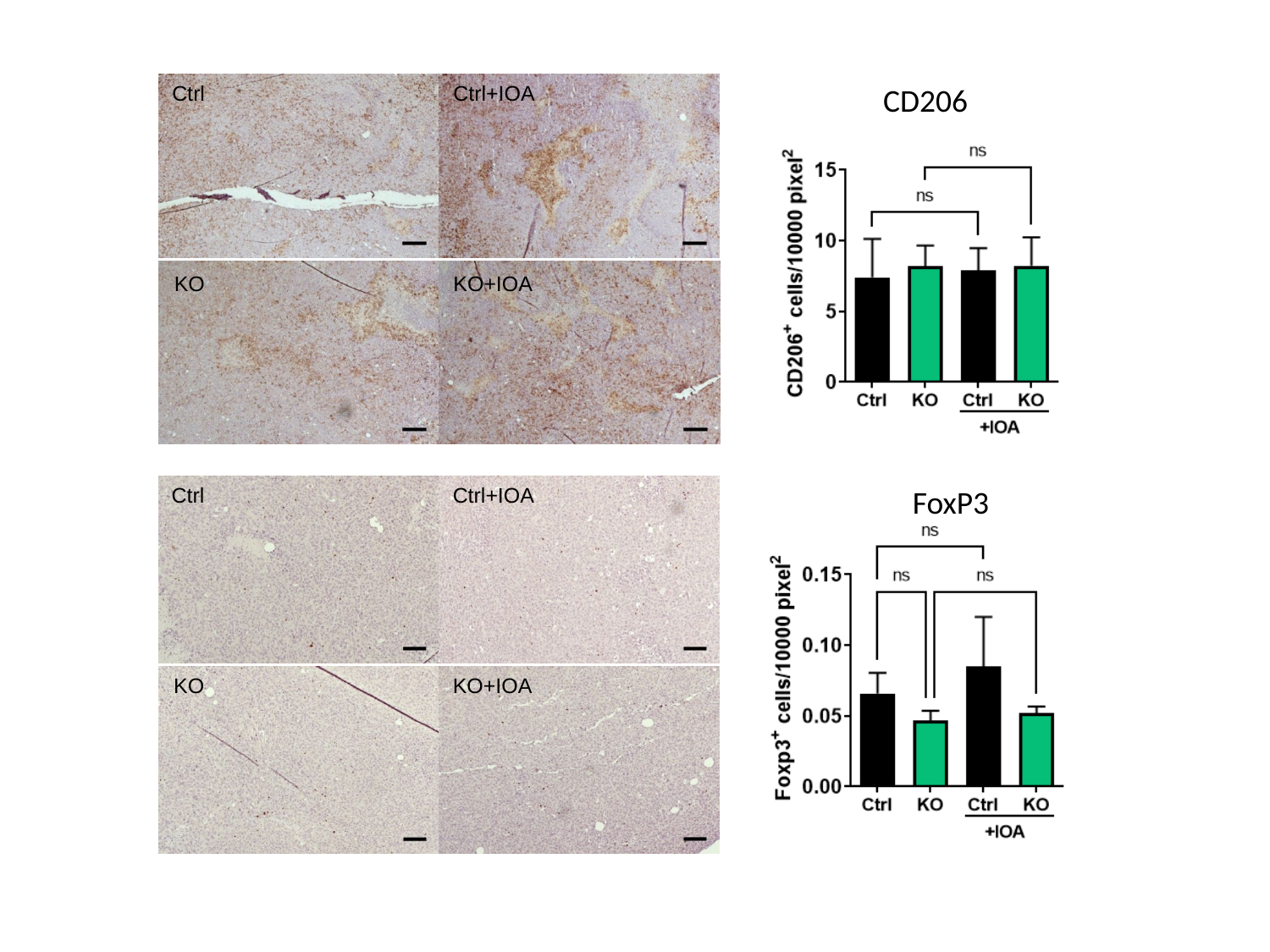

Ctrl
Ctrl+IOA
KO
KO+IOA
CD206
Ctrl
Ctrl+IOA
KO
KO+IOA
FoxP3

## Slide 9
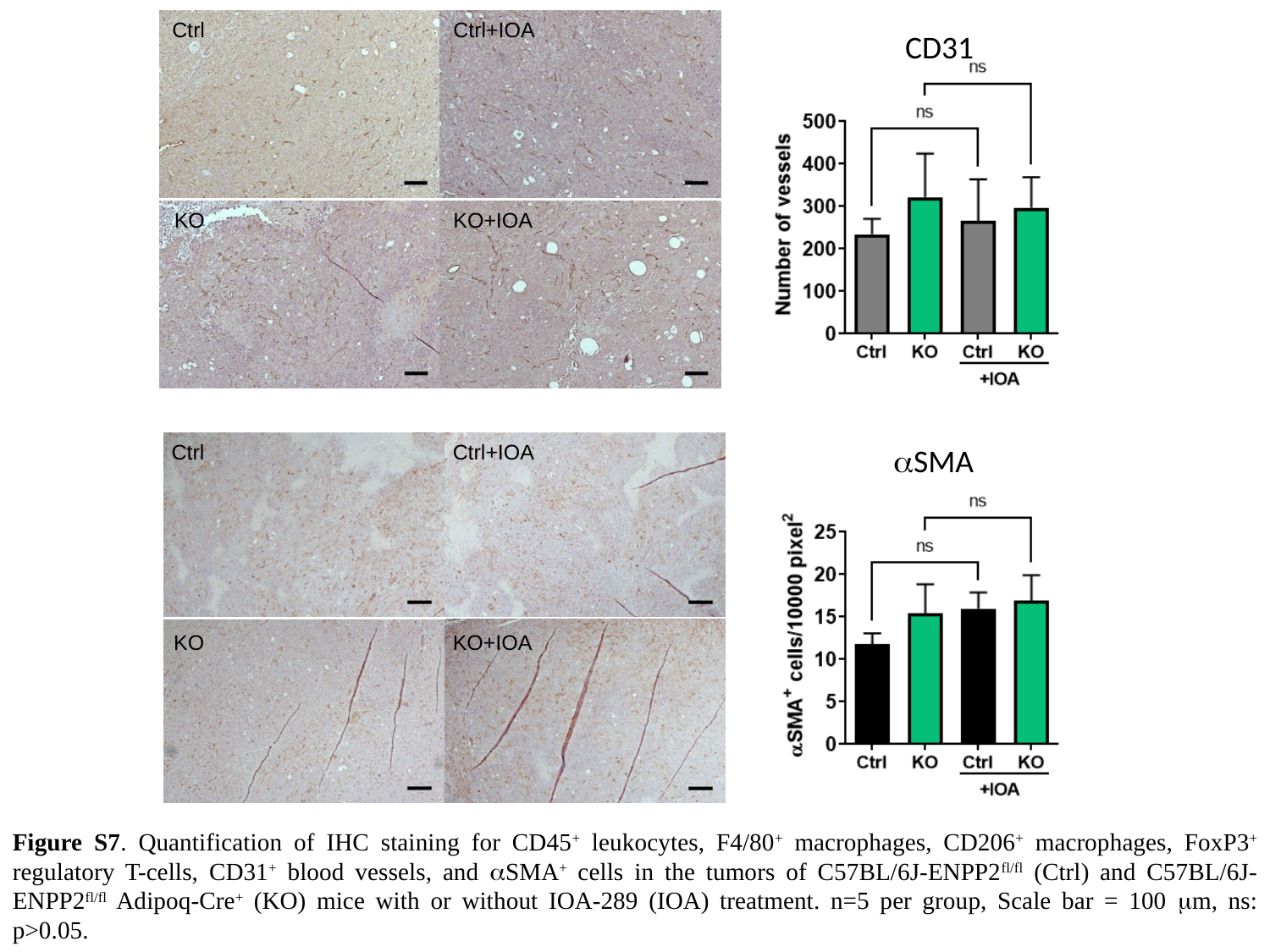

Ctrl
Ctrl+IOA
KO
KO+IOA
CD31
Ctrl
Ctrl+IOA
KO
KO+IOA
aSMA
Figure S7. Quantification of IHC staining for CD45+ leukocytes, F4/80+ macrophages, CD206+ macrophages, FoxP3+ regulatory T-cells, CD31+ blood vessels, and aSMA+ cells in the tumors of C57BL/6J-ENPP2fl/fl (Ctrl) and C57BL/6J-ENPP2fl/fl Adipoq-Cre+ (KO) mice with or without IOA-289 (IOA) treatment. n=5 per group, Scale bar = 100 mm, ns: p>0.05.

## Slide 10
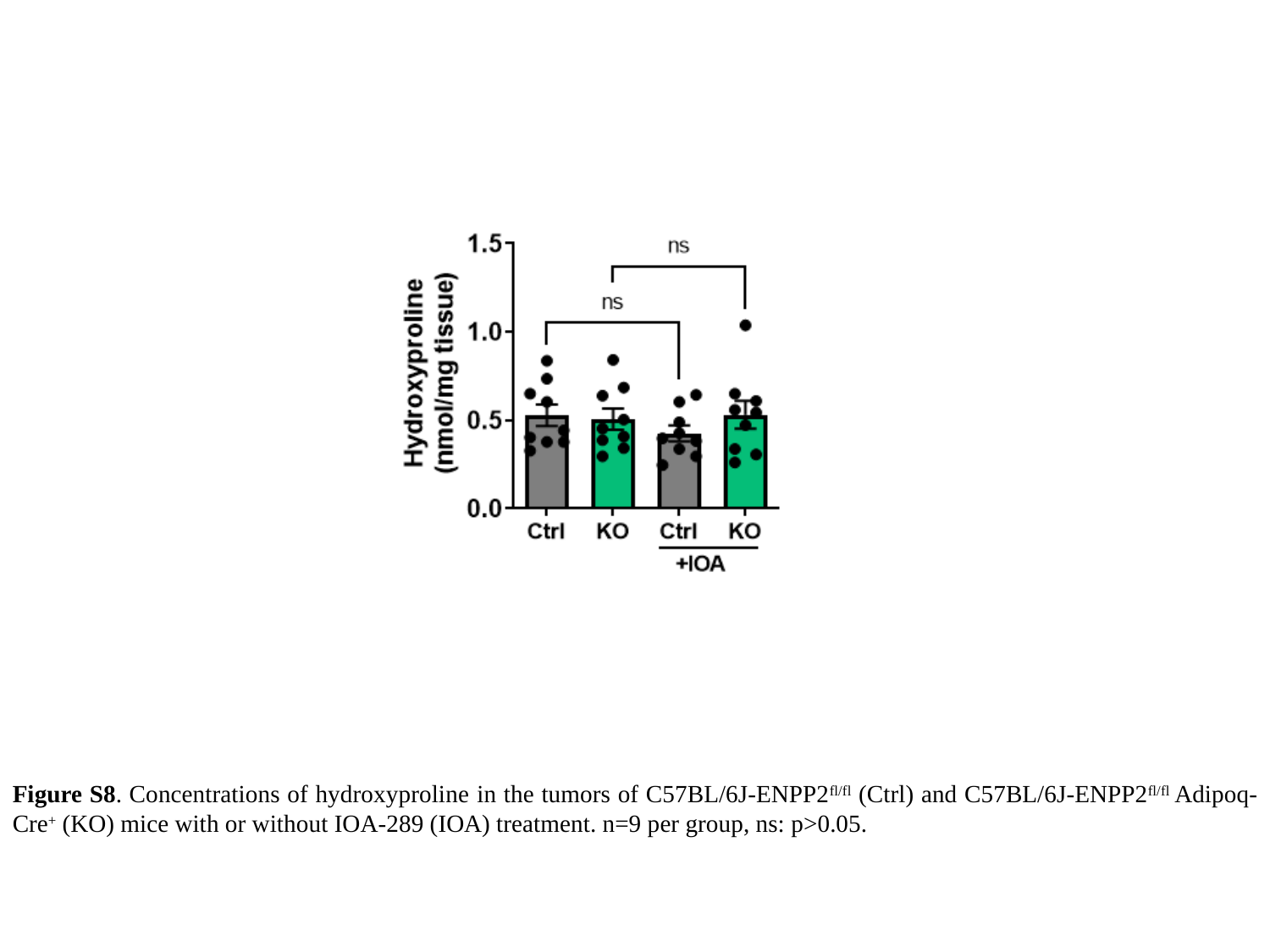

Figure S8. Concentrations of hydroxyproline in the tumors of C57BL/6J-ENPP2fl/fl (Ctrl) and C57BL/6J-ENPP2fl/fl Adipoq-Cre+ (KO) mice with or without IOA-289 (IOA) treatment. n=9 per group, ns: p>0.05.
